# Case presentation methods: a randomized controlled trial of the one-minute preceptor versus SNAPPS in a controlled setting

**DOI:** 10.1007/s40037-020-00588-y

**Published:** 2020-05-19

**Authors:** Eleonora D. T. Fagundes, Cássio C. Ibiapina, Cristina G. Alvim, Rachel A. F. Fernandes, Marco Antônio Carvalho-Filho, Paul L. P. Brand

**Affiliations:** 1 Department of Pediatrics, Federal University of Minas Gerais, Belo Horizonte, Brazil; 2 Department of Clinical Medicine, University of Campinas, Campinas, Brazil; 3 Center for Education Development and Research in Health Professions, University of Groningen and University Medical Center Groningen, Groningen, The Netherlands

**Keywords:** SNAPPS, One-minute preceptor, Case presentation, Clinical reasoning

## Abstract

**Introduction:**

One-minute preceptor (OMP) and SNAPPS (a mnemonic for Summarize history and findings; Narrow the differential; Analyze the differential; Probe the preceptor about uncertainties; Plan management; and Select case-related issues for self-study) are educational techniques developed to promote learners’ expression of clinical reasoning during the case presentation in the workplace. The aim of this present study was to compare the content of the case presentation between the SNAPPS and the OMP methods.

**Methods:**

This was a randomized controlled trial comparing SNAPPS and OMP in 60 medical students at the beginning of their fifth year of medical school. After an introduction session, students presented and discussed two cases based on real patients and provided in written format. All case presentations were recorded and evaluated by two researchers. The assessed elements of the case presentations were divided into three subgroups related to expression of clinical reasoning, time and initiative to guide the presentation.

**Results:**

There were 30 participants in each group. There was no difference in the expression of clinical reasoning between OMP and SNAPPS groups (number of differential diagnoses, justification of most likely diagnosis and differential diagnosis, expression of comparing and contrasting hypotheses). However, students in the SNAPPS group expressed significantly more questions and uncertainties (*p* < 0.001), and more often took the initiative to present and justify the most likely diagnosis, differential diagnosis and management plan than students in the OMP group, both in simple and complex cases (all *p* values <0.001) without extending the length of the teaching session.

**Conclusion:**

OMP and SNAPPS equally promote medical students’ expression of clinical reasoning. The SNAPPS technique was more effective than the OMP technique in helping students to take on an active role during case presentation. We propose SNAPPS as an effective learning tool, engaging students and promoting the expression of their clinical reasoning as part of a case presentation.

## Introduction

Clinical reasoning refers to the cognitive process that is necessary to evaluate and manage a patient’s medical problem [1]. It is a core competence of medical practice, which medical students and junior doctors need to learn under the supervision of clinical teachers. In everyday practice, the oral case presentation (‘tell me about the patient you just saw in clinic’) is the most commonly used tool allowing clinical teachers to evaluate and activate the learner’s clinical reasoning [2]. However, in such oral case presentations medical students and inexperienced junior doctors focus mainly on factual information and seldom express spontaneously their thoughts and reasoning, making assessment difficult [3, 4]. Despite their widespread use, oral case presentations have received little attention in the medical education literature [2]. There are not established methods and the formats derive partly from preceptor preferences but also depend on the learner and context. The lack of sufficient evidence about teaching methods in the clinical setting is a critical gap in the literature.

Two educational techniques have been developed to promote and assess learners’ clinical reasoning. One-minute preceptor (OMP) provides preceptors (clinical teachers) with five steps to guide the learner’s case presentation: get a commitment, probe for supporting evidence, teach general rules, reinforce what was right and correct mistakes [5]. The SNAPPS method is a mnemonic guiding the learner to structure the case presentation into six steps: Summarize history and finding; Narrow the differential; Analyze the differential; Probe the preceptor about uncertainties; Plan management; and Select case-related issues for self-study [6]. Whilst the OMP method gives instructions to teachers, SNAPPS provides them to learners. The latter approach has theoretical advantages because it helps learners to understand what is expected of them and take on a central role during the case presentation. Such active learning strategies support learner autonomy, foster motivation and, consequently, impact positively on the learning outcomes [7, 8]. Both OMP and SNAPPS encourage students’ and residents’ clinical reasoning and involve them in the patient management plan, reducing the likelihood of exchange of only factual information [5, 9–12]. Only one study to date has compared SNAPPS and OMP. No difference was found between the methods, except in the number of questions and uncertainties raised by residents, which was higher in the SNAPPS group [13].

The aim of the present study was to assess the differences between SNAPPS and OMP in terms of the content of case presentations by undergraduate students, using simple and more complicated pediatric cases. We hypothesized that students in the SNAPPS group would be more likely to express their clinical reasoning than students in the OMP group.

## Methods

### Setting and participants

This was a randomized controlled trial comparing SNAPPS and OMP in 60 medical students at the beginning of their fifth year of medical school at the Federal University of Minas Gerais, Brazil. This is a 6-year curriculum, comprising 18 months of preclinical content and 4.5 years of the clinical cycle, with the last 2 years in clinical clerkship rotations. Between March and May 2018, all participants were recruited during their first clerkship, which was in pediatrics and lasted 12 weeks. Before participating, students had had contact with real pediatric patients for approximately 4 h per week in the first 2.5 years of the clinical cycle in outpatient clinics in primary care. Participants were assigned by an assistant not otherwise involved in the study using simple randomization.

### Data collection

Students assigned to the SNAPPS group attended a 30-minute orientation session in which we demonstrated the SNAPPS technique and showed an example of a case presentation using SNAPPS on video, after which students had the opportunity to ask questions. They were given a pocket-sized card listing the six SNAPPS steps.

Students assigned to the OMP group attended a 30-minute session on the basics of clinical reasoning and case presentation feedback. Because case presentation with the OMP technique is guided by the teacher, no specific explanation of the method was given. However, they received information about what was expected of them while discussing a clinical case: a summary of the case, presentation of the most likely diagnosis, differential diagnosis and a management plan.

In the week following the introduction session, students presented and discussed two cases based on real patients and provided in written format with one pediatric preceptor (EDTF). The first case was simple enough to allow prompt diagnosis (pneumonia, viral upper respiratory tract infection, or gastroenteritis with mild dehydration). The second case was more complex to encourage clinical reasoning and express questions and uncertainties(neonatal cholestasis, acute abdominal pain or febrile newborn). Each case consisted of a description of a pediatric patient’s medical history with present complaints and findings from a physical examination. They had been developed by three teachers, experts in pediatrics. Three different cases were used in each category to minimize the exchange of case information between students. Each student received one simple and one complex case by random selection. All cases concerned diseases that the students were familiar with by prior teaching. All instructions about presentations were given with the case for both groups: students were asked to provide the most likely diagnosis, differential diagnosis and management plan for the case. There was no time restriction to read, prepare and present the case.

We audiotaped all case presentations. Two researchers (EDTF and CCI) independently assessed these audiotaped case discussions using a coding checklist, after a pilot study. Differences between the assessors were resolved by consensus. The assessed elements of the case presentations were divided into three subgroups related to:

- Expression of clinical reasoning: number of differential diagnoses, justification of most likely diagnosis and differential diagnosis (number of reported clinical data to explain a diagnosis, for example, fever, cough and tachypnea for pneumonia), expression of uncertainties to preceptor;
- Time (in minutes):
  - Total length of session: total duration of presentation and case discussion;
  - Summary of the case length: time spent only with the description of the case (history and physical examination) until the first question asked by the preceptor or the first report about the diagnosis expressed by the student;
  - Length of student’s speech during the discussion, after summary of the case: total student speech time, without teacher speech.
- Initiative to guide the presentation: learners’ initiatives to present and justify the most likely diagnosis and differential diagnosis, to present a plan for management in the way they had been instructed.

As secondary outcomes, we studied differences between groups related to making the right diagnosis and the management plan.

### Data analysis

Since there were no preliminary data studying the same outcome variables, the sample size calculation could only be based on assumptions. Assuming a desired power of 80% and an alpha of 0.05, a sample size of 30 students in each group would be able to detect a difference of at least 37% for quantitative variables and 35% for nominal variables, which we considered to be relevant and meaningful.

We used Pearson’s chi-square or Fisher’s exact test for categorical variables to compare outcomes between methods. Student’s t‑test was used to compare means between groups. We used parametric tests, even for variables with non-normal distributions because of the relatively large sample size and the lack of outliers. Levine’s test was used to verify the homogeneity of variance. To compare outcomes between simple and complex cases in each method, we used McNemar’s or Student’s test for paired nominal data.

The level of significance was <0.05 for all tests. IBM SPSS Statistics version 25 was used for statistical analysis.

### Ethical considerations

The study was approved by the Research Ethics Committee of Federal University of Minas Gerais (no. 82727518.8.0000.5149). All students participated voluntarily after providing written informed consent.

## Results

There were 30 participants in the SNAPPS group (16 men, 14 women; mean (SD) age 23.8 (2.1) years) and 30 in the OMP group (13 men, 17 women; age 23.3 (2.2) years). There was no difference related to age or sex between the groups (*p* = 0.34 and 0.438 respectively).

Tab. 1 shows the results for each outcome with the simple and complex case in the SNAPPS and OMP groups. There was no difference in the total length of the session between the SNAPPS and the OMP presentations. However, the length of students’ speech during the case presentation session was almost one minute longer in the SNAPPS group than in the OMP group, both with simple and complex cases.

**Table 1:** Outcomes for simple and complex cases from the SNAPPS and OMP group

Outcomes		Simple case			Complex case		
		SNAPPS	OMP	*p* value	SNAPPS	OMP	*p* value
Expressing clinical reasoning	Number of justifications for the most likely diagnosis	3.9 (1.5)	4.4 (1.6)	0.25	3.9 (1.0)	4.0 (1.4)	0.25
	Number of differential diagnoses presented	2.0 (0.9)	1.5 (1.3)	0.11	2.9 (1.3)	2.5 (1.2)	0.31
	Number of justification for differentials	3.4 (1.5)	2.9 (1.5)	0.24	3.8 (1.5)	3.7 (1.9)	0.80
	Number of questions and uncertainties presented	1.6 (0.8)	0.5 (0.8)	**<0.001**	1.7 (0.9)	0.9 (1.0)	**<0.001**
	Number of management plans presented	3.3 (1.0)	3.1 (0.9)	0.43	4.0 (1.3)	3.5 (1.3)	0.18
Time	Total meeting length (minutes)	7.6 (2.4)	7.4 (2.5)	0.59	9.1 (2.1)	8.8 (2.1)	0.73
	Summary of the case length (minutes)	1.9 (0.7)	2.1 (0.7)	0.23	2.2 (0.8)	2.3 (0.7)	0.51
	Student’s speech length during the discussion, after the summary of the case (minutes)	3.6 (1.5)	2.8 (1.4)	** 0.04**	4.2 (1.4)	3.3 (1.2)	** 0.010**
Taking initiative to	Present the most likely diagnosis *N* (%)	30 (100%)	7 (23.3%)	**<0.001**	30 (100%)	7 (23.3%)	**<0.001**
	Justify the most likely diagnosis *N* (%)	29 (96.7%)	20 (66.7%)	**<0.001**	29 (96.7%)	27 (90%)	0.61
	Present the differential diagnosis *N* (%)	30 (100%)	5 (16.7%)	**<0.001**	29 (96.7%)	17 (56.7%)	**<0.001**
	Justify the differential diagnosis *N* (%)	29/30 (96.7%)	21/23 (91.3%)^a^	0.57	30/30 (100%)	25/28 (89.3%)^a^	0.11^a^
	Initiate patient management plans *N* (%)	30 (100%)	9 (30%)	**<0.001**	29 (96.7%)	12 (40%)	**<0.001**
Setting the	Correct diagnosis *N* (%)	29 (96.7%)	28 (93.3%)	1.00	27 (90%)	23 (76.7%)	0.17
	Right management plan promptly *N* (%)	28 (93.3%)	21 (70%)	** 0.02**	29 (96.7%)	20 (66.7%)	**<0.001**

Data represent mean (SD) unless otherwise specified

^a^Only for students who present a differential diagnosis

There was no difference in expressing clinical reasoning assessed by the number of differential diagnoses, justifications for the most likely diagnosis and differential diagnosis. Students in the SNAPPS group expressed significantly more questions and uncertainties in both simple and complex cases than students in the OMP group (Tab. 1).

Students in the SNAPPS group more often took the initiative to present and justify the most likely diagnosis, the differential diagnosis and the management plan, both for the simple and complex cases (all *p* values <0.001), except in relation to justifying the most likely diagnosis in complex cases (*p* = 0.61).

There was no difference in making the correct diagnosis between the groups for either case. However, the proportion of students in the SNAPPS group who proposed the appropriate management plan was higher than that in the OMP group for simple (*p* = 0.020) and complex (*p* = 0.003) cases.

There was no difference in the performance of OMP and SNAPPS regarding simple vs complex cases.

## Discussion

The results from this study demonstrate that SNAPPS helped students to take on a more active role during case presentation than with the OMP method, initiating the discussion about diagnosis, expressing their clinical reasoning and planning the patient’s care without extending the length of the session. These outcomes have important implications for managing some of the main challenges of clinical teaching and learning: the relatively passive role of the learners, the exchange of only factual information without expression of clinical reasoning, and the supervisors’ high workload and perceived lack of supervision time [3, 4].

Case presentations require many skills including gathering and summarizing patient data, and elaboration and expression of clinical reasoning. It is a challenge to achieve such complex skills by just watching peers or clinical teachers in action, which is why supervisors should facilitate this process by clarifying instructions and guiding the learners through the steps of clinical reasoning [14]. OMP and SNAPSS were developed to help students in this process in different ways, allowing them to express their clinical reasoning. Such active learning strategies impact positively on the learning outcomes and academic performance [8]. In the present study, the outcomes related to the expression of clinical reasoning were not different between the OMP and SNAPPS groups, except in relation to the number of questions and uncertainties presented. Only one study to date has compared SNAPPS and OMP, using a single simulated case with a clear diagnosis, musculoskeletal lower back pain, with similar results in relation to the number of differential diagnoses and management plans [13].

However, students in the SNAPPS group initiated the discussion about diagnosis and management plan significantly more often than those from the OMP group, both in simple and complex cases (Tab. 1). Although students from both groups received instructions about what was expected during case presentation, OMP students were more likely to wait for teachers to prompt them on their clinical reasoning. Teachers with a high clinical workload may be less inclined to provide such guidance. Based on adult learning principles and encouraging autonomy, learners using the SNAPPS approach assume a key role during the session with their supervisors. The finding that students in the SNAPPS group spoke for almost one minute more than those in the OMP group (Tab. 1) suggests that SNAPPS allows learners to really lead the discussion on clinical reasoning instead of waiting for the clinical teacher’s questions. Taking such an active role in clinical practice is crucial to achieving the desired educational outcomes, enhancing one’s sense of competence [6, 8, 15]. Furthermore, engaging students in the patient’s care may booster the feeling of relatedness. Students can really feel part of the team. These are key elements of self-determination theory [8].

As expected, the number of questions and uncertainties presented was higher with both cases in the SNAPPS group than in the OMP group. This is in accordance with the only previous study comparing the two methods [13]. SNAPPS is structured to encourage learners to express their uncertainties. This raises learners’ awareness that they can and should ask questions, which boosts their confidence to express their clinical reasoning. In this way, SNAPPS is both a learning and an assessment tool, allowing teachers to detect gaps in trainees’ knowledge or in the process of clinical reasoning [6, 12]. In addition, SNAPPS helps teachers to identify learners’ doubts and uncertainties, allowing immediate and tailored feedback to help shape the medical students’ and junior doctors’ learning. In our study, students in the SNAPPS group more often proposed the correct management plan than those in the OMP group. It is likely that this is the result of SNAPPS allowing learners to clarify their uncertainties during the discussion of the case. In 2012, Wolpaw et al. performed a secondary analysis of the type of uncertainties students have and the nature of teachers’ responses. They demonstrated that preceptors responded with teaching aligned with these uncertainties [16]. The OMP method proposes a step of “teach general rules” related to the case, but this is more likely to address what teachers consider relevant than to address learners’ uncertainties.

In addition to the advantages related to promotion of active engagement of learners, SNAPPS also proved to be a time-efficient method. Preceptors often struggle with time constraints in clinical settings, particularly when having to address both patient care and teaching objectives when supervising a medical student or junior doctor. In our study, SNAPPS presentations were no longer than OMP presentations, even with complex cases. In previous studies, the case presentation length was no different between SNAPPS and the traditional supervisor method [10, 12, 17, 18].

The limitations of the present study include the use of written cases in a controlled setting, without the pressure and challenges of real clinical teaching. We created simple and complex cases to diversify and evaluate the outcomes in different situations, but we realize that simulated cases may not represent the complexity of real cases [19]. With real cases, students have to manage the process of gathering all relevant information from patients, another skill in a different competency domain, before the case presentation. Using real cases, Wolpaw et al. found that the SNAPPS group outperformed the outcomes when compared with a control group [5]. Further studies are needed to corroborate our results in the context of real clinical teaching and with learners of different expertise levels, such as less experienced students and residents, and to evaluate long-term outcomes related to clinical reasoning for both methods. Another limitation may be a performance bias because OMP students did not receive specific instructions about the method, as the technique was developed to be guided by the teacher. However, both groups of students received the same information about what was expected of them: a summary of the case, presentation of the most likely diagnosis, a differential diagnosis and a management plan. We therefore expected students in the OMP group to take the initiative to present the diagnosis and management plan as much as those in the SNAPPS group, but this was not the case. Furthermore the rater was not blinded because the methods are clearly different and it becomes evident when the student using SNAPPS leads the discussion rather than waiting for the teacher’s questions.

In the present study, students proved to be able to use the SNAPPS method after a single 30-minute training session. Thus, teaching SNAPPS to students appears to be simple, efficient, and time effective. In addition, SNAPPS achieved important short-term learning outcomes, with no extra effort from the teacher to lead the session. The SNAPPS method encourages a more active role by the learners. OMP also achieved important outcomes related to expression of clinical reasoning. Furthermore, beginner students may have more difficulties in managing different demands at the same time, such as gathering patient data, summarizing the case, elaborating the diagnosis and planning the patient’s care. In these cases, when closer guidance is needed, OMP might be the preferable method to help less experienced students to express their clinical reasoning and to engage in the patient’s care. With the progressive improvement of clinical competences, SNAPPS can help to better shape a more active role by the learners.

This study provides evidence to support the use of SNAPPS as a learning tool to promote clinical reasoning as part of a case presentation’s routine for medical students. Its utilization represents short but multiple teaching/learning opportunities, encouraging students to take on an active role and to engage in the patient’s care.
